# Supplementary material for: Suboptimal factors in maternal and newborn care for refugees: Lessons learned from perinatal audits in the Netherlands
Source: PLoS One. 2024 Jun 27;19(6):e0305764. doi: 10.1371/journal.pone.0305764 (PMC11210813; doi:10.1371/journal.pone.0305764)
Supplement: S1 File — (DOCX) [file pone.0305764.s001.docx]

**Appendix 1: National Perinatal Audit Registry in the Netherlands**

*This appendix is partially based on previous descriptions of the Dutch national perinatal audit by van der Geest et al. and Rosman et al.* [21], [22]*.*

During perinatal audit meetings, healthcare providers evaluate the provided care in cases with adverse perinatal or maternal outcomes. Audit meetings take place biannually at most hospitals in the Netherlands. Each audit meeting is prepared by a local team and chaired by an independent supervisor. All perinatal healthcare professionals within a region are invited to audit meetings, including obstetricians, community midwives, clinical midwives, pediatricians, and obstetric nurses. Cases are discussed anonymously and involved healthcare providers can stay anonymous if they wish.

Which cases are discussed depends on the cases healthcare providers submit and whether these cases fall within one of the four audit themes. These themes change every two to three years and are determined by Perined in collaboration with experts in the field. During this study, the audit themes included late premature mortality (between 32+0 and 36+6 weeks), perinatal asphyxia (above 37+0 weeks), hyperbilirubinemia, and uterine rupture. Most cases discussed in perinatal audits fall within these themes although exceptions to this rule exist.

During an audit meeting, suboptimal factors and improvement points are constructed based on healthcare providers' evaluation of the cases. After reaching a consensus on the formulation of suboptimal factors and action points, these are registered in the National Perinatal Audit Registry. The local audit team also constructs a chronological report of each discussed case, which includes maternal characteristics, obstetric history, relevant prenatal consultations, a delivery report, and a summary of postpartum care, including maternal and neonatal follow-up. As this report is based on medical records, its completeness depends on the accuracy and thoroughness of the involved healthcare professionals’ documentation.

Chronological reports and their corresponding suboptimal factors and action points are stored in the national Perinatal Audit registry. This registry facilitates the confidential and anonymous registration of the cases, the audit process, and its outcomes. As most local teams only discuss two cases per audit meeting, the National Perinatal Audit registry doesn’t contain all cases with adverse outcomes, but only the selection of cases discussed in local audits.

**Appendix 2: Detailed description of suboptimal factors**

Table A: Detailed description of suboptimal factors

| Suboptimal factor | Description in case report: | Specification or issues/consequences described |
| --- | --- | --- |
| Phase 1: Care seeking |  |  |
| Untimely start of antenatal care | First antenatal care appointment after 12 weeks of gestation. | Average start of antenatal care in weeks + days of pregnancy: 18+1 (range 12+4 - 33+4). |
| Missed appointments/ late arrival | One or more missed appointments with the midwife or in the hospital, or late arrival with consequences for care. |  |
| Non-compliance | Clients’ non-compliance with healthcare providers' advice. | Most important consequences:   - Missed or delayed diagnostics tests. - Medication not used according to prescription. - Refused induction of labor. |
| Delayed care seeking in case of alarm symptoms | More than two days delay in consulting healthcare provider or no consultation at all in the case of alarm symptoms or symptoms of labor. | Most common alarm symptoms with delay in care:   - Reduced fetal movements. - Stomach-aches. - Vaginal blood or fluid loss. |
| Vulnerable context | Factors in woman’s social situation that significantly impacted care-seeking behavior according to the care provider. | Most common factors:   - Teenage pregnancy. - Domestic violence. - Undocumented status. |
| Partially uncontrolled pregnancy | Interruption of antenatal care | N = 5 cases:   - Four cases of interrupted care for 10, 6, 4, and 7 weeks at the gestational age of 27, 32, 27, and 20 weeks respectively. - In one case with an unknown number of weeks of interrupted care, return to care at 36+6 weeks. |
| Lack of trust in healthcare providers | Cases in which patients' lack of trust was explicitly mentioned by the care provider |  |
| Phase 2: Accessibility of services |  |  |
| Language barrier | Language barrier with mention of consequences leading to suboptimal care | Consequences described:   - Inadequate/insufficient counseling for prenatal diagnostics and/or mode of birth. - Inadequate/insufficient coaching during labor. - Difficulties in interpreting patients’ symptoms. - Incomplete patient histories. - Unnecessary hospital admission. - Use of general anesthesia during an emergency cesarean. - No follow-up care postpartum provided. - Missed appointments. - Missed psychological screening. |
| Inadequate involvement of professional interpreters | At least one prenatal or post-partum consultation where:   1. Healthcare providers described insufficient involvement of a professional interpreter in the case report. 2. Negative consequences of a language barrier were described, but a professional interpreter was not involved. 3. No involvement of an interpreter was described in situations considered crucial for communication according to the research group, while it was described during other consultations. | Most mentioned informal interpreters, as a consequence of not working with professional interpreters:   - Clients’ partner. - Family member. - Friend. - Neighbor. - Underaged child. - An unknown man from the waiting room. |
| Transportation difficulties | Any description of problems related to the transportation of clients to care facilities. | Problems mentioned:   - No transportation available/accessible for women to reach the midwifery practice or hospital for consultations. - No transportation available/accessible during labor. - Taxi service for asylum seekers hard to reach, delayed service, or taxi divider's refusal to transport asylum seekers during labor.   Consequences of transportation difficulties:   - More home consultations. - Midwives take clients in their own cars. - Missed appointments. - Continued antenatal care with a community midwife while hospital care was indicated. - Delay in arrival at the hospital in a case with potentially life-threatening complications. |
| Transfer of care | Transfer of care between care facilities during pregnancy for reasons other than medical indication. | Reasons described:   - Relocations between asylum centers. - Women moving house. - Threatening deportation. - Living with different family members because of undocumented status. - Transfer of care on clients’ request.   Consequences of a transfer of care during pregnancy:   - Partially uncontrolled pregnancies. - Missed hospital appointments. - Missed or repeated diagnostic tests due to the incomplete or late transfer of medical records. |
| Financial barriers | Explicit mention of financial barriers to care | Consequences:   - No use of folic acid. - No uptake of postpartum care. - Not coming to appointments because of fear of costs. |
| The stress of the asylum procedure | Description of uncertainty or stress surrounding the asylum procedure | Examples:   - Asylum-seeking women with threat of deportation. - Fear of losing residence permit. |
| Phase 3: Quality of care |  |  |
| Missed or late diagnostic tests | Diagnostic tests should have been performed, however these opportunities were missed or carried out with a delay. | Most common diagnostics missed:   - Bilirubin testing after detection of neonatal jaundice. - Doppler and growth ultrasounds in case of IUGR. - Blood pressure measurements. - Fetal growth ultrasounds or CTGs in case of reduced fetal movements. - Fetal scalp blood sampling during labor. - Thompson scores and temperature management in case of perinatal asphyxia. - MRSA screening. - Diagnostics for persistent anemia. |
| Communication issues between care providers | Description or mention of communication issues between care providers or organizations involved in care for asylum seekers | Most common issues described:   - Incomplete or absent transfer of medical information between midwives and hospitals, different midwifery practices, different hospitals, midwives and general practitioners, and among staff within hospitals. - Late or absent communication from the Dutch Central Agency for the Reception of asylum seekers with regards to the deportation or relocation of asylum seekers. - Ultrasound centers or laboratories not communicating results with midwives. - Delay in consulting a university hospital in complex cases. - The lack of a case manager who keeps track of a client's care process. |
| No or late start of treatment | Treatment would have been indicated but was carried out late or not at all. | Most common examples:   - Delayed intervention during labor (most often in the form of an emergency cesarean). - Delayed phototherapy treatment of a neonate after detecting hyperbilirubinemia. - Missed opportunity for prophylactic treatment with acetylsalicylic acid, tranexamic acid, and/or calcium. - Delayed start of antibiotics in neonates with infection. |
| Other inadequate care | Inadequate care provided, not fitting into a different suboptimal factor | Most common issues described:   - Incorrect medication or incorrect dose of medication given. - Type of treatment obsolete/not evidence-based. - Care not according to a specific protocol for the condition. |
| Issues concerning documentation | Case report reflects issues such as incompleteness, inconsistency, or complete absence of documentation with a possible effect on care. | Most common examples:   - Inconsistent notes on case details or treatment provided. - Lack of notes on the treatment plan or care providers' considerations. - Lack of documentation regarding diagnostic examinations performed. |
| Incomplete history taking or counseling | Case report reflects incomplete history taking or counseling of the patient | Most common examples:   - No risk assessment of patient history for hyperbilirubinemia. - Incomplete medical history obtained. - No counseling concerning the mode of birth, risks of post-term birth, and induction of labor. - Insufficient counseling to ensure patients' understanding and trust. |
| Logistic or technical issues | Issues in logistics or availability of technical support | Most common issues:   - Staff short on time/high work pressure. - Shortage of staff, e.g., no gynecologist available to perform an emergency cesarean section. - No operation or labor room available. - No NICU availability. - Lab results unavailable. - Malfunctioning of CTG device. |
| Missed or late diagnosis | Medical conditions that were either missed or diagnosed late. | Missed diagnoses:   - Hyperbilirubinemia. - Uterus rupture. - IUGR. - Pre-eclampsia. - Neonatal coarctation aortae. - Anemia requiring blood transfusion. - Maternal acute fatty liver syndrome. |
| Delay in consultation or referral | The patient should have been referred or invited for an appointment after a referral | Most common issues:   - Delay between the registration of a patient at the midwifery clinic and intake consultation. - No referral for neonatal icterus on the first day after birth. - No referral to emergency care in case of alarm symptoms, such as severe vomiting in the third trimester, lack of fetal movements, and thrombocytopenia. |
| Insufficient or inadequate psychosocial care | Insufficient or inadequate care offered to clients with known psychological health problems | Most common examples include women for whom no referral to specialist care was made despite healthcare providers’ awareness of one of the following:   - Unsafe home situation due to domestic violence. - Mental health symptoms such as stress, depression, or anxiety. - Unwanted pregnancy. - Traumatic birth. |
| Inadequate action in case of no-show | Insufficient or inadequate action from the healthcare provider after multiple missed appointments without notice from the client | In all cases, this concerned missed appointments without action from healthcare providers, or actions were not as recommended by national guidelines on birth care for asylum seekers. |
| Insufficient monitoring during labor | Insufficient monitoring of a woman in labor or the fetal condition during labor | Most common examples:   - CTG not monitored regularly by a care provider. - Technical failure of CTG equipment. - No registration of maternal contractions. |
| Issues with postnatal maternity care | Insufficient attention of healthcare providers to counsel patients and/or make arrangements concerning postnatal maternity care | - Postnatal maternity care was arranged very late in pregnancy or not arranged at all. - Maternity care assistants not accepted by patients due to misunderstandings about the concept of maternity care. |
| Healthcare providers’ negative attitude | Negative attitudes towards clients reflected by the negative framing of patients in cases where other issues, such as communication difficulties, presumably played a role. | Examples of terms used to refer to patients by healthcare providers:   - ‘Uncooperative’ - 'Incapable of following instructions' - ‘Unreasonable’ - ‘Unmanageable’   In all cases, communication difficulties had been previously described, whereas no professional interpreters were involved. |
| Inadequate risk assessment | The responsible care provider should have been different based on the patient's known obstetric risk status | Most common issues:   - Care by a community midwife while hospital care was indicated. - Care in a regular hospital while care in a university hospital for complex conditions would have been indicated. |
| No placental pathology while indicated | Lack of diagnostics on placental pathology while indicated |  |

**Appendix 3: Suboptimal factors per adverse outcome**

Table A: Suboptimal factors per adverse outcome

| Suboptimal factors | Number of cases with suboptimal factors | | | | | |
| --- | --- | --- | --- | --- | --- | --- |
|  | Total | Intrauterine fetal death | Perinatal asphyxia above 37 weeks | Severe neonatal hyperbilirubinemia | Uterine Rupture | Other |
| **Total** | 53 | 14 | 15 | 12 | 7 | 7 |
| **Phase 1: Care seeking** | 43 (29+14) | 13 (7+6) | 10 (6+4) | 10 (7+3) | 6 (5+1) | 6 (6+0) |
| Untimely start of antenatal care | 22 (21+1) | 8 (7+1) | 6 (6+0) | 4 (4+0) | 4 (4+0) | 2 (2+0) |
| Missed appointments/ late arrival | 22 (19+3) | 5 (4+1) | 10 (9+1) | 5 (4+1) | 2 (2+0) | 1 (1+0) |
| Non-compliance | 20 (17+3) | 5 (4+1) | 7 (6+1) | 4 (3+1) | 2 (2+0) | 3 (3+0) |
| Misunderstanding | 10 (10+0) | 4 (0+0) | 4 (4+0) | 1 (1+0) | 2 (2+0) | 0 (0+0) |
| Patient’s choice | 2 (1+1) | 0 (0+0) | 2 (1+1) | 0 (0+0) | 0 (0+0) | 0 (0+0) |
| Un**clear** | 10 (8+2) | 2 (1+1) | 2 (2+0) | 3 (2+1) | 0 (0+0) | 3 (3+0) |
| Delayed care seeking in case of alarm symptoms | 18 (11+7) | 6 (2+4) | 4 (2+2) | 4 (4+0) | 3 (2+1) | 2 (2+0) |
| Vulnerable context | 15 (14+1) | 5 (5+0) | 3 (3+0) | 4 (3+1) | 3 (3+0) | 2 (2+0) |
| Partially uncontrolled pregnancy | 5 (5+0) | 0 (0+0) | 2 (2+0) | 1 (1+0) | 1 (1+0) | 1 (1+0) |
| Lack of trust in healthcare provider | 2 (1+1) | 1 (1+0) | 1 (0+1) | 0 (0+0) | 0 (0+0) | 0 (0+0) |
| **Phase 2: Accessibility of services** | 50 (42+8) | 13 (12+1) | 15 (13+2) | 10 (7+3) | 7 (4+3) | 7 (7+0) |
| Language barrier | 45 (38+7) | 9 (8+1) | 15 (13+2) | 9 (7+2) | 7 (4+3) | 7 (7+0) |
| Inadequate involvement of an official interpreter | 31 (24+7) | 7 (6+1) | 10 (8+2) | 5 (3+2) | 6 (3+3) | 5 (5+0) |
| Transportation difficulties | 12 (11+1) | 5 (5+0) | 1 (1+0) | 5 (4+1) | 0 (0+0) | 1 (1+0) |
| Transfer of care | 10 (10+0) | 2 (2+0) | 5 (5+0) | 2 (2+0) | 1 (1+0) | 1 (1+0) |
| Financial barriers | 3 (3+0) | 0 (0+0) | 0 (0+0) | 1 (1+0) | 1 (1+0) | 1 (1+0) |
| Uncertainty or stress surrounding the asylum procedure | 3 (3+0) | 2 (2+0) | 0 (0+0) | 1 (1+0) | 0 (0+0) | 0 (0+0) |
| **Phase 3: Quality of care** | 53 (24+29) | 14 (7+7) | 15 (9+6) | 12 (1+11) | 7 (2+5) | 7 (5+2) |
| Communication issues between care providers | 33 (29+4) | 8 (7+1) | 11 (11+0) | 6 (4+2) | 5 (5+0) | 4 (3+1) |
| Missed, late, or incomplete diagnostic tests | 32 (20+12) | 10 (7+3) | 7 (6+1) | 7 (0+7) | 6 (5+1) | 2 (2+0) |
| Late diagnostics after detecting neonatal jaundice | 7 (0+7) | NA | NA | 7 (0+7) | NA | NA |
| Other inadequate care | 26 (21+5) | 8 (7+1) | 4 (4+0) | 6 (3+3) | 6 (4+2) | 4 (4+0) |
| No or late start of treatment | 24 (10+14) | 6 (3+3) | 8 (3+5) | 4 (1+3) | 4 (1+3) | 3 (2+1) |
| Incomplete history taking or counseling | 24 (17+7) | 4 (4+0) | 4 (3+1) | 12 (6+6) | 4 (4+0) | 1 (1+0) |
| Issues concerning documentation | 19 (19+0) | 8 (8+0) | 5 (5+0) | 2 (2+0) | 3 (3+0) | 2 (2+0) |
| Missed or late diagnosis | 18 (5+13) | 6 (2+4) | 2 (1+1) | 6 (1+5) | 5 (2+3) | 1 (0+1) |
| Logistic or technical issues | 16 (14+2) | 2 (1+1) | 6 (5+1) | 5 (5+0) | 3 (3+0) | 1 (1+0) |
| Delay in consultation or referral | 16 (9+7) | 5 (4+1) | 2 (2+0) | 7 (2+5) | 2 (2+0) | 2 (1+1) |
| Insufficient or inadequate psychosocial care | 14 (14+0) | 5 (5+0) | 3 (3+0) | 3 (3+0) | 3 (3+0) | 1 (1+0) |
| Inadequate action in case of no-show | 8 (8+0) | 2 (2+0) | 2 (2+0) | 4 (4+0) | 0 (0+0) | 0 (0+0) |
| Healthcare providers’ negative attitude | 8 (8+0) | 2 (2+0) | 4 (4+0) | 0 (0+0) | 3 (3+0) | 0 (0+0) |
| Insufficient monitoring during labor | 7 (5+2) | 1 (1+0) | 5 (3+2) | 0 (0+0) | 2 (2+0) | 1 (1+0) |
| Issues with postnatal maternity care | 6 (4+2) | 0 (0+0) | 1 (1+0) | 5 (3+2) | 0 (0+0) | 0 (0+0) |
| Inadequate risk assessment | 4 (3+1) | 2 (1+1) | 1 (1+0) | 0 (0+0) | 0 (0+0) | 1 (1+0) |
| No placental pathology while indicated | 4 (4+0) | 0 (0+0) | 2 (2+0) | 0 (0+0) | 1 (1+0) | 1 (1+0) |

Numbers are presented as: Number of cases (minor+major).
