# Supplementary material for: Suboptimal factors in maternal and newborn care for refugees: Lessons learned from perinatal audits in the Netherlands
Source: PLoS One. 2024 Jun 27;19(6):e0305764. doi: 10.1371/journal.pone.0305764 (PMC11210813; doi:10.1371/journal.pone.0305764)
Supplement: Attachment — Submitted filename: Verschuuren et al. manuscript.docx [file pone.0305764.s002.docx]

# Suboptimal factors in maternal and newborn care for refugees: lessons learned from perinatal audits in the Netherlands.

A.E.H. Verschuuren, MD, J.B. Tankink, MD, I.R. Postma MD/PhD, K.A. Bergman MD, B. Goodarzi, RM PhD, E.I. Feijen-de Jong, RM PhD, J.J.H.M. Erwich, MD/PhD

A.E.H. Verschuuren, MD, [a.e.h.verschuuren@umcg.nl](mailto:a.e.h.verschuuren@umcg.nl) (Corresponding author)

University Medical Centre Groningen & University of Groningen, Department of Health Sciences, Global Health Unit, Hanzeplein 1, 9713 GZ Groningen, the Netherlands.

ORCID: 0000-0002-8954-4850

J.B. Tankink, MD

Erasmus University Medical Centre, Department of Obstetrics and Gynaecology, Doctor Molewaterplein 40, 3015 GD Rotterdam, The Netherlands.

ORCID: 0009-0006-0994-2194

I.R. Postma, MD PhD

Medical Center Leeuwarden, Department of Obstetrics and Gynecology, Leeuwarden, the Netherlands.

University Medical Centre Groningen & University of Groningen, Department of Health Sciences, Global Health Unit, Groningen, the Netherlands.

ORCID: 0000-0001-6312-376X

K.A. Bergman MD

University Medical Centre Groningen & University of Groningen, Department of Paediatrics Beatrix Children’s Hospital, Hanzeplein 1, 9713 GZ Groningen, the Netherlands.

B. Goodarzi, RM PhD

Amsterdam UMC location Vrije Universiteit Amsterdam, department of Midwifery Science, de Boelelaan 117, Amsterdam, The Netherlands

University Medical Center Groningen & University of Groningen, Department of Primary care and Longterm Care, PO Box 196, 9700 AD, Groningen, the Netherlands

Amsterdam Public Health, Quality of Care, Amsterdam, The Netherlands

Midwifery Academy Amsterdam Groningen, InHolland, Amsterdam, The Netherlands

ORCID: 0000-0002-9758-1299

E.I. Feijen-de Jong, RM PhD

University Medical Centre Groningen & University of Groningen, Department of Primary care and Longterm Care, PO Box 196, 9700 AD, Groningen, the Netherlands

Amsterdam UMC location Vrije Universiteit Amsterdam, department of Midwifery Science, De Boelelaan 1117, Amsterdam, the Netherlands

Amsterdam Public Health, Quality of Care, Amsterdam, the Netherlands

Midwifery Academy Amsterdam Groningen, InHolland, Groningen, the Netherlands

ORCID: 0000-0001-5766-296X

Prof. J.J.H.M. Erwich, MD/PhD

University Medical Centre Groningen & University of Groningen, Department of Obstetrics and Gynecology, Hanzeplein 1, 9713 GZ Groningen, the Netherlands.

ORCID: 0000-0003-1362-4501

# ABSTRACT

**Introduction:** Refugees and their health care providers face many challenges in receiving and providing maternal and newborn care. Research exploring how these challenges are related to adverse perinatal and maternal outcomes is scarce. Therefore, this study aims to identify suboptimal factors in maternal and newborn care for asylum-seeking and refugee women and assess how often they contribute to adverse pregnancy outcomes in the Netherlands.

**Methods:** We conducted a retrospective audit of 53 cases with adverse perinatal and maternal outcomes in women with a refugee background. Suboptimal factors in care were identified and categorized according to Binder et al.’s Three Delays Model and their relationship with the adverse outcome was assessed.

**Results:** 29 categories of suboptimal factors were identified, of which seven were related to care seeking (1^st^ delay), six to the accessibility of services (2^nd^ delay), and 16 to quality of care (3^rd^ delay). All 53 cases contained suboptimal factors, and in 67.9% of cases at least one of these factors most likely or probably contributed to the adverse perinatal or maternal outcome.

**Conclusion:** This study demonstrated that suboptimal factors in maternal and newborn care for refugee women contribute to adverse perinatal and maternal outcomes. This suggests that some adverse outcomes in refugee populations could be avoided if care was better adjusted to women’s needs. These results urge health care providers and policymakers to adjust not only the maternal and newborn care system for refugees but also the refugee system to achieve health care equity. Necessary adjustments include culturally sensitive education for health care providers, increased workforce diversity, minimizing the relocation of asylum seekers, and permanent reimbursement of professional interpreter costs.

**Keywords:** Refugees; Pregnancy; Culturally competent care; Adverse Birth Outcomes; Prenatal care; Postnatal care

# INTRODUCTION

The rise of forced migration worldwide urges maternal and newborn care providers to respond to the needs of pregnant refugees^[1]^ and their children (1). In the Netherlands, every year approximately 600 babies are born to mothers living in asylum seeker centers. The number of babies born to refugee women^[2]^ with a residence permit is likely larger, but the number remains unknown (2). A substantial body of international literature has demonstrated that asylum seekers and refugee women with a residence permit have poorer perinatal and maternal outcomes compared to non-migrant populations, including higher rates of perinatal and maternal mortality and morbidity (2–6).

Given these disparities, access to high-quality maternal and newborn care is essential to promote the health and well-being of pregnant refugees. However, these women face many barriers in accessing maternal and newborn care, such as linguistic differences, a disadvantaged socio-economic status, racial, ethnic, and cultural discrimination, limited knowledge of the host country’s health care system, and the stress of resettlement in a new country (7–9). Professionals also face multiple challenges in providing care to refugees (10,11). A previous study from the Netherlands identified five categories of challenges community care midwives face while providing care for refugee women, including interdisciplinary collaboration, communication with clients, continuity of care, psychosocial care, and the vulnerable context of clients (12).

Given these challenges, suboptimal care may contribute to perinatal health inequities between refugee and non-migrant populations. However, research exploring to what extent these challenges affect care and which factors contribute to adverse perinatal and maternal outcomes in refugee populations is scarce. Therefore, this study aims to identify suboptimal factors in maternal and newborn care for refugee women and assess how often they contribute to adverse pregnancy outcomes in the Netherlands.

# METHODS

Design

We conducted a retrospective audit of cases from the Dutch National Perinatal Audit registry, which concerned adverse perinatal and maternal outcomes in refugee women over a 3-year period (2017-2019).

The Perinatal Audit registry

Local perinatal audits are evaluations of severe cases of maternal or perinatal morbidity or mortality. These audits take place in all maternal and newborn care centers in the Netherlands. During an audit meeting, involved health care professionals systematically review case reports and identify improvement and action points for practice. For every case discussed, an individual case report is stored, which contains case characteristics and details about the provided perinatal care. Which cases are discussed depends on the cases health care providers submit and whether these cases fall within one of the four audit themes. During this study, the audit themes included late premature mortality (between 32+0 and 36+6 weeks), perinatal asphyxia (above 37+0 weeks), hyperbilirubinemia, and uterine rupture. The Dutch National Perinatal Audit registry contains all cases discussed in regional perinatal audits in the Netherlands (13). A more detailed explanation of the Perinatal Audit registry is included in Appendix 1.

Theoretical framework: The Three Delays Model

In 1994, Thaddeus and Maine proposed the Three Delays Model to facilitate the identification of factors that cause a delay in care and might therefore contribute to adverse outcomes (14). The model identifies three phases of possible delay. Phase 1 (‘care seeking’) involves all factors that influence a woman’s decision to seek emergency or non-emergency care. Phase 2 (‘accessibility of services’) factors reflect a woman’s ability to identify and reach an appropriate medical facility. Lastly, phase 3 (‘quality of care’) comprises factors that allow a woman to receive optimal care once the facility has been reached. The model was originally designed for low-resource settings but was modified by Binder et al. to evaluate maternal and newborn care for migrant populations in high-income settings (15). In this study, we apply the modified version of the model.

Study population

We included all cases from the Dutch National Perinatal Audit registry that concerned refugees. These cases included asylum-seeking women, refugee women with residence permits, and undocumented women, altogether referred to as ‘refugees’ in this study.

Between March and May 2021, the first author systematically hand-searched all individual reports to identify refugee women, because migration history and legal status are not included in the Perinatal Audit registry's standard administration. Cases were included based on the mother's country of origin or if terminology in the case report suggested a refugee background (see box 1). We included countries of origin based on the absolute number of asylum applications and the percentage of immigrants that apply for asylum from these countries according to Statistics Netherlands (16). Cases were excluded if women had lived in the Netherlands for more than ten years, if there was uncertainty about a woman’s migration background, or if no case report could be retrieved from the Perinatal Audit registry.

Box 1: Words, phrases, and countries of origin, identified in cases, which made them eligible for inclusion.

Words or phrases which made cases eligible for inclusion:

Asylum seeker; Asylum seekers center; Refugee camp; Refugee status; Residence permit; Fled from; Dutch Council for Refugees; GCA/GZA*; Temporary residence in the Netherlands due to political tensions back home.

Countries of origin eligible for inclusion:

Syria; Somalia; Iran; Eritrea; Afghanistan; Pakistan; Middle east; Congo; Ethiopia; Turkey**; Ghana; Nigeria;

* Organization that provides primary health care for asylum seekers in the Netherlands.

** Only eligible for inclusion in combination with another factor such as a short stay in the Netherlands

Identification and classification of suboptimal factors

After case selection, data was extracted in a three-step process. Initially, suboptimal factors were identified, then categorized and ultimately classified according to the three delays model. For the identification of suboptimal factors, all included reports were independently reviewed by the first two authors (AV and JT) and the co-authors, including a midwife (EF-dJ), an obstetrician (JE), an obstetrician in training (IP), and a neonatologist (KB). The individual descriptions of suboptimal care were combined by the first author (AV). Cases in which there was no consensus on the descriptions of suboptimal care were discussed in a meeting with the entire team and conflicts were resolved. The experts considered care suboptimal for patient-related factors (phases one and two of the Three Delays Model) if they negatively affected the care refugee women received with a possible negative effect on the outcome. Quality-related factors (phase three of the Three Delays Model) were considered suboptimal if care deviated from the professional requirements of standard care, national guidelines, or local protocols.

For the categorization of suboptimal factors, suboptimal care descriptions were qualitatively coded and categorized by two researchers (AV and JT). An inductive analysis was performed to identify suboptimal factor categories using Atlas ti. Version 8.4.5. These factors were classified according to Binder et al’s Three Delays Model (15).

We conducted this analysis in addition to the original local audit as our objective was to include patient-related factors that were not incorporated in original audits. During all stages of the analysis, the research team was blinded to the suboptimal factors identified in original local audits. If the original local audit contained suboptimal factors not identified by the research team, these were added after classification. Descriptive statistics on suboptimal factors were performed using SPSS version 28.0.0.0.

## Minor/Major analysis

For each suboptimal factor, the research team assessed to which extent it was associated with the adverse outcomes. Factors were initially assessed by the first author (AV) and checked by one or two members of the expert team according to their expertise. Factors were labeled ‘minor’ if any contribution to the adverse outcome was unlikely or uncertain. If suboptimal factors most likely or probably contributed to the adverse outcome, factors were labeled ‘major’.

Ethics

This study was assessed by the medical ethical committee of the University Medical Centre Groningen (METc 2021/375) and was not subject to the Medical Research Involving Human Subjects Act in the Netherlands. Regarding privacy issues, all data were retrieved and handled anonymously.

# RESULTS

Case selection

53 (4.7%) of the 1117 cases stored in the national Perinatal Audit concerned a refugee and are therefore included in the study. Of these 53 cases, 20 cases (37.7%) were included based on terminology in the case report that suggests a refugee background. 33 (62.3%) cases were included because they concern women from countries that suggest a background of forced migration (see box 1).

The audit processes

Seven cases were discussed with all members of the research team per request of one of the team members when in doubt whether a factor should be classified as suboptimal. The first author discussed ten additional cases in the team as there was an incongruity in the expert’s description of suboptimal care. In a three-hour long meeting, all members of the team discussed seventeen cases and two general issues, which included the definition of an untimely start of antenatal care and what to do with missing data in case reports. In this meeting, all discrepancies and unclarities between experts were resolved. After the expert team reached a consensus, descriptions of suboptimal care were combined to form 29 different categories of suboptimal factors presented in this study. A detailed description of suboptimal factors is included in Appendix 2.

The original local audits identified 119 suboptimal factors, divided over 43 cases (10 cases contained no suboptimal factors in the Perinatal Audit Registry). These factors were assigned to the 29 suboptimal factor categories and all factors corresponded to our framework. Of the factors identified in the original local audits, 45.4% (n=54) addressed the same suboptimal factor identified by the research team, while 45.4% (n=54) were not identified by the research team. 8.4% (n=10) of suboptimal factors were not included because they concerned team evaluations or peer support for health care providers (n=4), it was unclear what specific suboptimal factor they targeted (n=3) or it was unclear what was meant (n=3).

Study population

Case characteristics are summarized in Table 1. Refugee women were born in the Middle East, Africa, or Eastern Europe. At the start of pregnancy care, 24.5% of women were asylum seekers and 22.6% were refugees with a residence permit. In the rest of the cases (50.9%), women’s residence status was missing from the perinatal audit data.

Table 1: Case characteristics

| Case characteristics | Total (N=53) |
| --- | --- |
| Origin of the mother |  |
| Middle East* | 26 (49.1) |
| Africa^†^ | 20 (37.7) |
| Eastern-Europe^‡^ | 3 (5.7) |
| Unknown | 4 (7.5) |
| Residence status at the start of pregnancy care |  |
| Asylum seeker | 13 (24.5) |
| Refugee with a residence permit | 12 (22.6) |
| Unknown | 28 (52.8) |
| Duration of stay in the Netherlands (years) |  |
| < 1 year | 18 (34.0) |
| < 2 years | 6 (11.3) |
| 3 - 4 years | 9 (17.0) |
| 4 - 10 years | 6 (11.3) |
| Unknown | 14 (26.4) |
| Age of the mother |  |
| < 20 | 4 (7.5) |
| 20 - 29 | 20 (37.7) |
| 30 - 39 | 26 (49.1) |
| 40+ | 3 (5.7) |
| Parity |  |
| Nulliparous | 15 (28.3) |
| Multiparous (1,2,3) | 32 (60.4) |
| Grand multipara (≥4) | 6 (11.3) |

*Data are presented as: Number of cases (%)*

** Middle Eastern countries included: Syria, Iran, Iraq, Afghanistan, Pakistan, and Turkey*

*† African countries included: Somalia, Eritrea, Nigeria, Ethiopia, Congo, Ghana, Sudan, and Gambia*

*‡ Eastern European countries included: Bosnia, Belarus, and Moldavia*

Adverse outcomes

Adverse outcomes from cases are divided into five categories: fetal death (n=14), perinatal asphyxia (n=15), severe neonatal hyperbilirubinemia (n=12), uterine rupture (n=7), and other (n=7). The category ‘other’ includes neonatal mortality (n=2), postpartum hemorrhage (n=2), pre-eclampsia (n=1), meconium aspiration syndrome (n=1), GBS-sepsis (n=1) and one case in which a woman suffered from pre-eclampsia, placental rupture, and postpartum hemorrhage (n=1). The number of suboptimal factors per adverse outcome is included in Appendix 3.

Suboptimal factors

Of the 29 substandard factor categories, most factors (n=16, 55.2%) are included in phase three, while phase one contains seven (24.1%) and phase two contains six (20.7%) suboptimal factors. In most cases (n=36, 67.9%), at least one suboptimal factor is labeled major.

Table 2 describes the number of cases with suboptimal factors per adverse outcome. All cases contain at least one suboptimal factor in phase three and most cases also contain suboptimal factors in phases one and two. Phase three contains the most cases with major factors, followed by phase one and then phase two. The number of cases with major suboptimal factors in phase three is especially high in cases of severe neonatal hyperbilirubinemia (n=11, 91.7%).

Table 2: The number of cases with suboptimal factors per adverse outcome

| Adverse perinatal or maternal outcome | Number of cases with suboptimal factors | | | |  |
| --- | --- | --- | --- | --- | --- |
|  | Total* | Phase 1: Care seeking | Phase 2: Accessibility of services | Phase 3: Quality of care | |
| Intrauterine fetal death | 14 | 13 (7/6) | 13 (12/1) | 14 (7/7) | |
| Perinatal asphyxia** | 15 | 10 (6/4) | 15 (13/2) | 15 (9/6) | |
| Severe neonatal hyperbilirubinemia | 12 | 10 (7/3) | 10 (7/3) | 12 (1/11) | |
| Uterine Rupture | 7 | 6 (5/1) | 7 (4/3) | 7 (2/5) | |
| Other | 7 | 6 (6/0) | 7 (7/0) | 7 (5/2) | |

Numbers are presented as: Number of cases (minor/major).

*The total number of cases in this table is 55 because in two cases two adverse outcomes occurred. This includes one case in which uterine rupture led to perinatal asphyxia and one case in which fetal death was described and uterine rupture occurred during induced labor.

**Above 37 weeks of gestation

Phase one: care seeking

Suboptimal factors with a possible effect on care seeking occur in 43 cases (81.1%), and in 14 cases (26.4%) at least one of these factors is major. The most common suboptimal factors are an untimely start of antenatal care, missed or late arrival at appointments, and non-compliance (see Table 3). Of all suboptimal factors in phase one, delayed care seeking in case of alarm symptoms is most often related to adverse outcomes (n=7, 13.2%). Case A presents an example, in which major contributing factors in phase 1 are missed appointments and delayed care seeking in case of alarm symptoms. In phase two, a language barrier and inadequate involvement of an official interpreter are major suboptimal factors. The major factor identified in phase three is missed or late diagnostic tests.

Case A:

A young multiparous mother from the Middle East, who has been in the Netherlands as an asylum seeker for less than a year, frequently misses appointments throughout her pregnancy. Due to miscommunication, the patient misses blood glucose measurements and doesn’t go to a lab appointment her midwife had scheduled her for. A language barrier is mentioned by care providers as the reason for miscommunication and the patient’s missed appointments. At 32 weeks of gestation, the patient is referred to the hospital because her community care midwife suspects fetal growth restriction. Due to another miscommunication, the patient does not show up at the ultrasound appointment in the hospital. After three weeks, her midwife arranges a new appointment, and fetal growth restriction is diagnosed. The obstetrician decides that the fetal growth ultrasound must be repeated after two weeks, even though an additional ultrasound for doppler-flow measurements after one week would have been indicated according to Dutch care guidelines. More than two weeks later, with no record of a new fetal growth ultrasound, the patient’s partner phones the hospital with signs of labor. After arrival at the hospital, no fetal heartbeat is found, and fetal death is diagnosed. When asked, the patient reports that she hadn’t felt any fetal movements in the two days before the hospital visit.

Table 3: Suboptimal factors and their association with adverse outcomes grouped by phase of delay.

| Suboptimal factors | Number of cases with suboptimal factors | | |
| --- | --- | --- | --- |
|  | Total | Minor | Major |
| **Total** | 53 |  |  |
| **Phase 1: Care seeking** | 43 (81.1) | 29 | 14 |
| Untimely start of antenatal care | 22 (41.5) | 21 | 1 |
| Missed appointments/late arrival | 22 (41.5) | 19 | 3 |
| Non-compliance | 20 (37.7) | 17 | 3 |
| Misunderstanding | 10 | 10 | 0 |
| Patient’s choice | 2 | 1 | 1 |
| Unclear | 10 | 8 | 2 |
| Delayed care seeking in case of alarm symptoms | 18 (34.0) | 11 | 7 |
| Vulnerable context | 15 (28.3) | 14 | 1 |
| Partially uncontrolled pregnancy | 5 (9.4) | 5 | 0 |
| Lack of trust in health care provider | 2 (3.8) | 1 | 1 |
| **Phase 2: Accessibility of services** | 50 (94.3) | 42 | 8 |
| Language barrier | 45 (84.9) | 38 | 7 |
| Inadequate involvement of an official interpreter | 31 (58.5) | 24 | 7 |
| Transportation difficulties | 12 (22.6) | 11 | 1 |
| Transfer of care | 10 (18.9) | 10 | 0 |
| Financial barriers | 3 (5.7) | 3 | 0 |
| Uncertainty or stress surrounding the asylum procedure | 3 (5.7) | 3 | 0 |
| **Phase 3: Quality of care** | 53 (100) | 24 | 29 |
| Communication issues between care providers | 33 (62.3) | 29 | 4 |
| Missed or late diagnostic tests | 33 (62.3) | 20 | 13 |
| Other inadequate care | 25 (47.2) | 21 | 4 |
| No or late start of treatment | 24 (45.3) | 10 | 14 |
| Incomplete history taking or counseling | 24 (45.3) | 17 | 7 |
| Issues concerning documentation | 19 (35.8) | 19 | 0 |
| Missed or late diagnosis | 18 (34.0) | 5 | 13 |
| Logistic or technical issues | 16 (30.2) | 14 | 2 |
| Delay in consultation or referral | 16 (30.2) | 9 | 7 |
| Insufficient or inadequate psychosocial care | 14 (26.4) | 14 | 0 |
| Inadequate action in case of no-show | 8 (15.1) | 8 | 0 |
| Health care providers’ negative attitude | 8 (15.1) | 8 | 0 |
| Insufficient monitoring during labor | 7 (13.2) | 5 | 2 |
| Issues with postnatal maternity care | 6 (11.3) | 4 | 2 |
| Inadequate risk assessment | 4 (7.5) | 3 | 1 |
| No placental pathology while indicated | 4 (7.5) | 4 | 0 |

A detailed description of suboptimal factors can be found in Appendix 2

Phase two: accessibility of care

Suboptimal factors with a possible effect on the accessibility of care occur in 50 cases (94.3%), and in eight cases (15.1%) at least one of these factors is major. The most common suboptimal factors for refugee women while accessing perinatal care are language barriers and inadequate involvement of an official interpreter (see Table 3). In seven cases (13.2%), these factors are related to an adverse outcome. Case B presents an example, in which the major contributing factor for phase one concerns missed appointments. The major contributing factors in phase two are a language barrier and inadequate involvement of an official interpreter and in phase three this is an issue with postnatal maternity care.

Case B:

A primiparous woman from Afrika, who has been in the Netherlands for a little over a year, has an uncomplicated pregnancy when labor starts after 41 weeks of gestation. The patient does not speak any Dutch or English. Her partner serves as an interpreter, but his Dutch language skills are limited. After a difficult labor, complicated by shoulder dystocia, the patient gives birth to a child with a suboptimal start who recovers quickly (Apgar score of 8 after 1 minute, and 9 after 5 minutes). That same evening at nine pm, the family is discharged from the hospital and maternity care services are called for a home visit. As the concept of maternity care services was not sufficiently explained to the woman and her partner, they are asleep and have their phones turned off when the maternity care assistant rings the door of their home that evening and the next morning. When the community midwife arrives later that day, she discovers that the newborn’s temperature had not been monitored, there have been no hot water bottles in the baby’s bed, and breastfeeding had not yet succeeded. Moreover, the baby looks yellow. The midwife immediately arranges admission to the hospital, where treatment with phototherapy is started for hyperbilirubinemia.

Phase three: quality of care

Suboptimal factors with a possible effect on quality of care occur in 53 cases (100%), and in 29 cases (54.7%) at least one of these factors is major. Suboptimal factors which most often have a major association with adverse outcomes are no or late start of treatment (n=14, 26.4%), diagnosis (n=13, 24.5%), and diagnostic tests (n=13, 24.5%). As a part of missed or late diagnostic tests, late diagnostics after detecting neonatal jaundice occurs in seven cases and is always assessed as major. Communication issues between care providers is the most common suboptimal factor (n=33, 62.3%), although its relation to the outcome is often minor (n=29, 54.7%). A negative attitude by health care providers in case reports is observed in eight cases and includes describing patients as ‘incapable of following instructions', 'unreasonable', 'uncooperative', and 'unmanageable'. In all these cases communication issues have been described during pregnancy. Case C presents an example in which all suboptimal factors in phases one and two are deemed minor. Phase three is assessed major for incomplete history taking, late diagnostic tests, missed diagnosis, and a delay in referral.

Case C:

A primiparous woman from the Middle East has been in the Netherlands as an asylum seeker for around 6 months. She has an uncomplicated pregnancy and labor during which she gives birth without complications. During pregnancy, health care providers do not assess risk factors for neonatal hyperbilirubinemia while family history would have uncovered a high risk. Two days postpartum, the baby’s skin and eyes look yellow, and it has lost 9% of its birth weight. Two days later, even though the child has gained 80 grams, it is still yellow, and the maternity care assistant discovers urate crystals in the urine. No action is undertaken by any of the care providers. Seven days postpartum, the mother expresses worry because her baby hasn’t defecated for three days and seems less alert. The midwife immediately refers the family to the hospital where the baby is diagnosed with severe hyperbilirubinemia (bilirubin: 389 umol/l) and treated with phototherapy.

# DISCUSSION

In this perinatal audit study of 53 cases with adverse perinatal outcomes among refugees, we identified 29 categories of suboptimal factors in maternity care. Seven of these categories relate to care seeking, six to the accessibility of services, and sixteen to quality of care. All cases contain multiple suboptimal factors, and in two-thirds of these cases at least one factor most likely or probably contributed to the adverse perinatal or maternal outcome.

Most of the suboptimal factors in maternal and newborn care for refugee women identified in this study have been previously reported (4,5,23–29,10,12,17–22). Previous studies show that inadequate maternity care is more prevalent among refugee women compared to host country populations (24,25). In our study, suboptimal factors in maternal and newborn care contributed to adverse perinatal and maternal outcomes among refugees. Our findings in this context thus imply that suboptimal factors in maternal and newborn care play a role in perinatal health inequities between refugee and non-refugee populations, highlighting the need for targeted interventions in this area. The wide range of suboptimal factors identified in this study and their association with adverse outcomes challenge the Dutch healthcare system's fundamental principles of access to care, equity, and high-quality services for all (30). Moreover, forced migration into the Netherlands has a long-standing history, and health inequities have disadvantaged various migrant populations for decades (31,32). The subsequent paragraphs will discuss suboptimal factors identified in this study per phase of delay in care and provide recommendations on how to address these factors to improve maternal and newborn care for refugees.

## Phase 1: Care-seeking

The suboptimal factors related to care seeking in this study, emphasize the importance of promoting and facilitating care-seeking behavior among refugee women. It is important to recognize that limited care-seeking and perceived non-compliance are not solely attributable to refugees, but frequently stem from structural barriers on the individual, health care service, and migration policy levels (33). These barriers include but are not limited to, socio-economic disadvantage, unwelcoming attitudes towards refugees in the host country, health care providers’ lack of cultural competence, women's unfamiliarity with the health care system, and previous negative experiences of care (5,19,33–35). To improve refugee women's access to care, it is crucial to acknowledge that addressing individual behaviors alone is insufficient and that interventions must also target underlying structural barriers (36). Further research should explore the extent to which different structural barriers affect refugee women’s access to care and identify best practices in this regard. Further research and interventions should be developed and implemented by policymakers and health care providers in collaboration with refugees (37–40).

Phase 2: Accessibility of services

This study adds to a large body of evidence demonstrating the harmful impact of unaddressed language barriers in health care (5,18,19). In many of the audited cases, official interpreters were not routinely involved, and care providers commonly relied on women’s language skills, nonverbal communication, or informal interpreters. These alternative communication strategies limit women's ability to understand medical information and compromise the safety, confidentiality, and accuracy of translations (41,42). Barriers to language support can be a direct consequence of political choices. For instance, in our study period community care midwives in the Netherlands were unable to claim the costs of professional interpreter services for refugees with a residence permit. This stresses the need for the permanent reimbursement of interpreter costs in all refugee receiving countries, which the Dutch government reinstalled in maternal and newborn care as of January 2023 (43). Although studies show that the presence of professional interpreters improves clinical outcomes and patient satisfaction with care, professional interpreters alone do not resolve all communication barriers (41,44,45). Other factors, such as cultural differences, women’s experiences of discrimination or stigma, and broken trust between refugee women and healthcare providers, also influence communication in maternal and newborn care for refugees (5,12,35). Thus, to overcome communication barriers, efforts towards an inclusive health care system should be made, which encompass culturally sensitive care that considers the unique perspectives and practices of individuals from diverse backgrounds, such as refugees (46). One of the initial steps in realizing this objective is to provide culturally sensitive training and education for health care providers, including both current and aspiring professionals (47–50).

Other factors leading to phase 2 delays, such as transfer of care and stress surrounding the asylum procedure, illustrate how migration policy and the asylum seeker context compromise women’s ability to access care (51). Transfer of care often occurs due to the relocation of asylum seekers and leads to partially uncontrolled pregnancies, missed appointments, and missed or repeated diagnostic tests. These findings add to a growing body of evidence on the negative effects of relocations on the well-being of pregnant asylum seekers as well as the continuity and quality of care (4,12,20–23). This calls for an adjustment to the Dutch refugee system which limits the number of relocations for all asylum seekers, especially during pregnancy.

Phase 3: Quality of care

While several suboptimal factors observed in phase 3 have been reported in non-refugee populations, we also identified factors that appear more specific to refugees. These concern incomplete history taking or inadequate counseling, particularly regarding prenatal diagnostics, and issues with post-partum care, such as delayed arrangement (10,24–29,52,53). Furthermore, in contrast with previous audit studies that did not focus on refugees specifically, our findings present evidence for negative attitudes among health care providers in care for refugee women (10,24–29). Previous research on refugee women’s experiences and health care staff’s attitudes shows that racial and ethnic discrimination in care is common (5,54–56). This is concerning, as racism adversely affects the quality of care refugee women receive and is associated with a lack of trust and delayed care seeking (57–66). In many cases, health care providers may be unaware of their discriminatory behavior, as it may result from unconscious bias, prejudices, or stereotyping (67). Further research is necessary to better understand how implicit bias and discrimination affect the quality of maternity care provided to refugee populations in the Netherlands. In addition, efforts should be made to increase workforce diversity, as cohorts of both current and training health care providers are often not representative of the populations they serve (68,69). This is of fundamental importance as workforce diversity improves the cultural sensitivity of care and is associated with improved patient satisfaction and better communication between patients and their health care providers (48,49,68–71).

**Strengths and limitations**

The amount of detail in which suboptimal factors were described and the assessment of their relation to adverse outcomes pose major strengths of this study. In addition, the involvement of experts from all care professions involved in maternal and newborn care for refugees and the unanimous consensus reached on suboptimal factors by the expert team strengthen the validity of our conclusions.

The main limitation is that the Perinatal Audit registry only includes cases discussed in local audits and is therefore incomplete as not all cases with adverse outcomes are discussed during an audit. Due to the explorative scope of the study, we decided not to compare suboptimal factors between refugee and non-refugee populations, which limits conclusions on population-specific factors that influence care. In addition, reports stored in the Perinatal Audit registry contain summaries of patient records, which can make it challenging to distinguish inadequate documentation from actual instances of suboptimal care. To tackle this problem, we did not assign a suboptimal factor if there was any unclarity on whether or how care was provided. This probably explains why 45.4% of the suboptimal factors identified in the original local audits were not identified by our research team. Our outcomes therefore most likely reflect an underrepresentation of suboptimal factors in the study population.

# CONCLUSION

Suboptimal factors in maternal and newborn care for refugees contribute to adverse perinatal and maternal outcomes among refugee women. This finding implies that adverse outcomes in refugee populations are partially preventable if care would be better adjusted to women’s needs. The range of suboptimal factors identified in this study provides considerable scope for improvement of maternal and newborn care for refugee populations. This includes adjustments to both the maternal and newborn care system and the refugee system in all refugee receiving countries, such as culturally sensitive education for health care providers, increased workforce diversity, minimizing the relocation of asylum seekers, and permanent reimbursement of professional interpreter costs. Further research should focus on initiatives that address structural barriers in women’s access to care, alternative care strategies, and the extent to which implicit bias and discrimination contribute to adverse outcomes.

# DECLARATIONS

## Acknowledgments

The authors wish to thank A. Schonewille-Rosman from Perined, who enabled the audit database search and approved the final manuscript for publication.

## Conflicts of Interest

The authors declare no conflict of interest. The sponsors had no role in the design, execution, interpretation, or writing of the study.

## Funding

This study was supported by the research fund of the medical center Leeuwarden, the Netherlands. Grant number: 2021-13.

## Data availability statement

Restrictions apply to the availability of these data. Data was obtained from Perined, which is a Dutch organization that facilitates the national perinatal registry and audit databases. Therefore, upon reasonable request the authors can provide data on the suboptimal factors they identified in the audit cases with Perined’s permission, but they cannot share the actual audit reports.

## Ethical approval

This study was reviewed by the acknowledged medical ethical committee of the university medical center Groningen (METc 2021/375) and was not subject to the Medical Research Involving Human Subjects Act in the Netherlands.

# REFERENCES

1.	United Nations High Commissioner for Refugees (UNHCR). Global trends: forced displacement in 2021. 2022.

2.	Tankink JB, Verschuuren AEH, Postma IR, van der Lans PJA, de Graaf JP, Stekelenburg J, et al. Childbirths and the Prevalence of Potential Risk Factors for Adverse Perinatal Outcomes among Asylum Seekers in The Netherlands: A Five-Year Cross-Sectional Study. Int J Environ Res Public Health. 2021 Dec 8;18(24):12933. doi: 10.3390/ijerph182412933.

3.	Gieles NC, Tankink JB, Midde M van, Duker J, Lans P Van Der, Wessels CM, et al. Maternal and perinatal outcomes of asylum seekers and undocumented migrants in Europe: a systematic review. Eur J Public Health. 2019 Aug 1;29(4):714–23. doi: 10.1093/eurpub/ckz042.

4.	Verschuuren AEH, Postma IR, Riksen ZM, Nott RL, Feijen-de Jong EI, Stekelenburg J. Pregnancy outcomes in asylum seekers in the North of the Netherlands: a retrospective documentary analysis. BMC Pregnancy Childbirth. 2020 Dec 25;20(1):320. doi: 10.1186/s12884-020-02985-x.

5.	Heslehurst N, Brown H, Pemu A, Coleman H, Rankin J. Perinatal health outcomes and care among asylum seekers and refugees: a systematic review of systematic reviews. BMC Med. 2018 Jun;16(1):89. doi: 10.1186/s12916-018-1064-0.

6.	van Oostrum IEA, Goosen S, Uitenbroek DG, Koppenaal H, Stronks K. Mortality and causes of death among asylum seekers in the Netherlands, 2002-2005. J Epidemiol Community Heal. 2011;65(4):376–83. doi: 10.1136/jech.2009.087064.

7.	McKnight P, Goodwin L, Kenyon S. A systematic review of asylum-seeking women’s views and experiences of UK maternity care. Midwifery. 2019 Oct;77:16–23. doi: 10.1016/j.midw.2019.06.007.

8.	Machado MC, Fernandes A, Padilla B, Dias S, Gomes I, Dias A, et al. Maternal and child healthcare for immigrant populations. International Organization for Migration (IOM). Geneva, Switserland; 2009.

9.	Khanlou N, Haque N, Skinner A, Mantini A, Kurtz Landy C. Scoping Review on Maternal Health among Immigrant and Refugee Women in Canada: Prenatal, Intrapartum, and Postnatal Care. J Pregnancy. 2017;2017:1–14. doi: 10.1155/2017/8783294.

10.	Esscher A, Binder-Finnema P, Bødker B, Högberg U, Mulic-Lutvica A, Essén B. Suboptimal care and maternal mortality among foreign-born women in Sweden: maternal death audit with application of the ‘migration three delays’ model. BMC Pregnancy Childbirth. 2014 Dec 12;14(1):141. doi: 10.1186/1471-2393-14-141.

11.	Balaam MC, Kingdon C, Thomson G, Finlayson K, Downe S. “We make them feel special”: The experiences of voluntary sector workers supporting asylum seeking and refugee women during pregnancy and early motherhood. Midwifery. 2016;34:133–40. doi: 10.1016/j.midw.2015.12.003.

12.	Verschuuren AEH, Tankink JB, Franx A, van der Lans PJA, Erwich JJHM, Feijen-de Jong EI, et al. Community midwives’ perspectives on perinatal care for asylum seekers and refugees in the Netherlands: a survey study. [Manuscript Submitted Publication]

13.	Eskes M, Waelput AJM, Erwich JJHM, Brouwers HAA, Ravelli ACJ, Achterberg PW, et al. Term perinatal mortality audit in the Netherlands 2010–2012: a population-based cohort study. BMJ Open. 2014 Oct 14;4(10):e005652. doi: 10.1136/bmjopen-2014-005652.

14.	Thaddeus S, Maine D. Too far to walk: maternal mortality in context. Soc Sci Med. 1994 Apr;38(8):1091–110. doi: 10.1016/0277-9536(94)90226-7.

15.	Binder P, Johnsdotter S, Essén B. Conceptualising the prevention of adverse obstetric outcomes among immigrants using the ‘three delays’ framework in a high-income context. Soc Sci Med. 2012 Dec;75(11):2028–36. doi: 10.1016/j.socscimed.2012.08.010.

16.	Statistics Netherlands. Asielverzoeken en nareizigers; nationaliteit, geslacht en leeftijd [Internet]. 30 november 2022. 2018 [cited 2019 May 7]. Available from: https://opendata.cbs.nl/statline/#/CBS/nl/dataset/83102NED/table?ts=1529128026717

17.	Till SR, Everetts D, Haas DM. Incentives for increasing prenatal care use by women in order to improve maternal and neonatal outcomes. Cochrane Database Syst Rev. 2015 Dec 15;2015(12). doi: 10.1002/14651858.CD009916.pub2.

18.	Bains S, Sundby J, Lindskog B V., Vangen S, Diep LM, Owe KM, et al. Satisfaction with maternity care among recent migrants: an interview questionnaire-based study. BMJ Open. 2021 Jul 16;11(7):e048077. doi: 10.1136/bmjopen-2020-048077.

19.	Cheng I-H, Drillich A, Schattner P. Refugee experiences of general practice in countries of resettlement: a literature review. Br J Gen Pract. 2015 Mar;65(632):e171–6. doi: 10.3399/bjgp15X683977.

20.	Tobin C, Murphy-Lawless. Irish midwives’ experiences of providing maternity care to non-Irish women seeking asylum. Int J Womens Health. 2014 Jan;159. doi: 10.2147/IJWH.S45579.

21.	Feldman R, Action M, Council R. When maternity doesn’t matter: dispersing pregnant women seeking asylum. Reprod Health Matters. 2013;21(42):212–7. doi: 10.1016/S0968-8080(13)42721-0.

22.	Chitongo S, Pezaro S, Fyle J, Suthers F, Allan H. Midwives’ insights in relation to the common barriers in providing effective perinatal care to women from ethnic minority groups with ‘high risk’ pregnancies: A qualitative study. Women and Birth. 2022 Mar;35(2):152–9. doi: 10.1016/j.wombi.2021.05.005.

23.	Fair F, Raben L, Watson H, Vivilaki V, van den Muijsenbergh M, Soltani H. Migrant women’s experiences of pregnancy, childbirth and maternity care in European countries: A systematic review. Daoud N, editor. PLoS One. 2020 Feb 11;15(2):e0228378. doi: 10.1371/journal.pone.0228378.

24.	Improving the health care of pregnant refugee and migrant women and newborn children. Copenhagen: WHO Regional Office for Europe; 2018.

25.	Sheikh J, Allotey J, Kew T, Fernández-Félix BM, Zamora J, Khalil A, et al. Effects of race and ethnicity on perinatal outcomes in high-income and upper-middle-income countries: an individual participant data meta-analysis of 2 198 655 pregnancies. Lancet. 2022 Dec;400(10368):2049–62. doi: 10.1016/S0140-6736(22)01191-6.

26.	Rosman AN, van Dillen J, Zwart J, Overtoom E, Schaap T, Bloemenkamp K, et al. Lessons learned from the perinatal audit of uterine rupture in the Netherlands: A mixed‐method study. Heal Sci Reports. 2022 Sep 4;5(5). doi: 10.1002/hsr2.664.

27.	van der Geest BAM, Rosman AN, Bergman KA, Smit BJ, Dijk PH, Been J V, et al. Severe neonatal hyperbilirubinaemia: lessons learnt from a national perinatal audit. Arch Dis Child - Fetal Neonatal Ed. 2022 Sep;107(5):527–32. doi: 10.1136/archdischild-2021-322891.

28.	de Reu P, Diem M Van, Eskes M, Oosterbaan H, Smits L, Merkus H, et al. The Dutch Perinatal Audit Project: a feasibility study for nationwide perinatal audit in the Netherlands. Acta Obstet Gynecol Scand. 2009 Nov;88(11):1201–8. doi: 10.3109/00016340903280990.

29.	Wolleswinkel-Van Den Bosch JH, Vredevoogd CB, Borkent-Polet M, Van Eyck J, Fetter WPF, Largo-Janssen TLM, et al. Substandard factors in perinatal care in The Netherlands: Acta Obstet Gynecol Scand. 2002 Jan;81(1):17–24. doi: 10.1034/j.1600-0412.2002.810104.x.

30.	Ministery of Health Welfare and Sport. Healthcare in the Netherlands. 2016.

31.	UNHCR’s Refugee Population Statistics Database [Internet]. [cited 2023 Mar 4]. Available from: https://www.unhcr.org/refugee-statistics/

32.	Carballo M, Grocutt M, Hadzihasanovic A. Women and migration: a public health issue. World Health Stat Q. 1996;49(2):158–64.

33.	Parajuli J, Horey D. Barriers to and facilitators of health services utilisation by refugees in resettlement countries: an overview of systematic reviews. Aust Heal Rev. 2020;44(1):132. doi: 10.1071/AH18108.

34.	Zeidan A, Khatri U, Munyikwa M, Barden A, Samuels-Kalow M. Barriers to Accessing Acute Care for Newly Arrived Refugees. West J Emerg Med. 2019 Oct 16;20(6):842–50. doi: 10.5811/westjem.2019.8.43129.

35.	Binder P. The Maternal Migration Effect: Exploring Maternal Healthcare in Diaspora Using Qualitative Proxies for Medical Anthropology. Uppsala university; 2012.

36.	Asif Z, Kienzler H. Structural barriers to refugee, asylum seeker and undocumented migrant healthcare access. Perceptions of Doctors of the World caseworkers in the UK. SSM - Ment Heal. 2022 Dec;2:100088. doi: 10.1016/j.ssmmh.2022.100088.

37.	Sandoval JA, Lucero J, Oetzel J, Avila M, Belone L, Mau M, et al. Process and outcome constructs for evaluating community-based participatory research projects: a matrix of existing measures. Health Educ Res. 2012 Aug 1;27(4):680–90. doi: 10.1093/her/cyr087.

38.	Baum F. Participatory action research. J Epidemiol Community Heal. 2006 Oct 1;60(10):854–7. doi: 10.1136/jech.2004.028662.

39.	Minkler M, Baden CA. Community-Based Participatory Research for Health: From Process to Outcomes. 2nd ed. John Wiley & Sons, Inc.; 2008. 243–261 p.

40.	Israel BA, , Eng E, Schulz A, Parker EA. Methods in Community-Based Participatory Research for Health. John Wiley & Sons, Inc.; 2013. 4–37 p.

41.	Patel P, Bernays S, Dolan H, Muscat DM, Trevena L. Communication Experiences in Primary Healthcare with Refugees and Asylum Seekers: A Literature Review and Narrative Synthesis. Int J Environ Res Public Health. 2021 Feb 4;18(4):1469. doi: 10.3390/ijerph18041469.

42.	Kasper A, Mohwinkel LM , Nowak AC, Kolip P. Maternal health care for refugee women - A qualitative review. Midwifery. 2022 Jan;104:103157. doi: 10.1016/j.midw.2021.103157.

43.	The Dutch Healthcare Authority (Nederlandse zorg autoriteit). Reimbursement of interpreters now possible in community midwifery care (Dutch: Declaratie talentolk nu mogelijk in eerstelijns geboortezorg) [Internet]. [cited 2023 Apr 7]. Available from: https://www.nza.nl/actueel/nieuws/2022/10/04/declaratie-talentolk-nu-mogelijk-in-eerstelijns-geboortezorg

44.	MacFarlane A, Huschke S, Pottie K, Hauck FR, Griswold K, Harris MF. Barriers to the use of trained interpreters in consultations with refugees in four resettlement countries: a qualitative analysis using normalisation process theory. BMC Fam Pract. 2020 Dec 5;21(1):259. doi: 10.1016/j.midw.2021.103157.

45.	Karliner LS, Jacobs EA, Chen AH, Mutha S. Do Professional Interpreters Improve Clinical Care for Patients with Limited English Proficiency? A Systematic Review of the Literature. Health Serv Res. 2007 Apr;42(2):727–54. doi: 10.1111/j.1475-6773.2006.00629.x.

46.	Lau LS, Rodgers G. Cultural Competence in Refugee Service Settings: A Scoping Review. Heal Equity. 2021 Mar 1;5(1):124–34. doi: 10.1089/heq.2020.0094.

47.	Lee A, Padilla C. Causes of health inequities. Curr Opin Anaesthesiol. 2022 Jun;35(3):278–84. doi: 10.1097/ACO.0000000000001142.

48.	Vance AJ, Bell T. Becoming an Antiracist Neonatal Community. Adv Neonatal Care. 2021 Feb;21(1):9–15. doi: 10.1097/ANC.0000000000000829.

49.	Smith DG. Building Institutional Capacity for Diversity and Inclusion in Academic Medicine. Acad Med. 2012 Nov;87(11):1511–5. doi: 10.1097/ACM.0b013e31826d30d5.

50.	Vella E, White VM, Livingston P. Does cultural competence training for health professionals impact culturally and linguistically diverse patient outcomes? A systematic review of the literature. Nurse Educ Today. 2022 Nov;118:105500. doi: 10.1016/j.nedt.2022.105500.

51.	Villalonga-Olives E, Kawachi I, von Steinbüchel N. Pregnancy and Birth Outcomes Among Immigrant Women in the US and Europe: A Systematic Review. J Immigr Minor Heal. 2017 Dec 24;19(6):1469–87. doi: 10.1007/s10903-016-0483-2.

52.	Koopmanschap I, Martin L, Gitsels - van der Wal J, Suurmond J. Counselling for prenatal anomaly screening to migrant women in the Netherlands: An interview study of primary care midwives’ perceived barriers with client–midwife communication. Eur J Midwifery. 2022 May 19;6(May):1–9. doi: 10.18332/ejm/147911.

53.	Posthumus AG, Peters IA, Borsboom GJ, Knapen MFCM, Bonsel GJ. Inequalities in uptake of prenatal screening according to ethnicity and socio-economic status in the four largest cities of the Netherlands (2011-2013). Prenat Diagn. 2017 Oct;37(10):959–67. doi: 10.1002/pd.5089.

54.	Ajjarapu A, Story WT, Haugsdal M. Addressing Obstetric Health Disparities among Refugee Populations: Training the Next Generation of Culturally Humble OB/GYN Medical Providers. Teach Learn Med. 2021 May 27;33(3):326–33. doi: 10.1080/10401334.2020.1813585.

55.	Hamed S, Bradby H, Ahlberg BM, Thapar-Björkert S. Racism in healthcare: a scoping review. BMC Public Health. 2022 May 16;22(1):988. doi: 10.1186/s12889-022-13122-y.

56.	Zemouri C. Discriminatie maakt ziek. Hoe patiënten met een migratieachtergrond door zorgdiscriminatie verslechterde zorg ontvangen. Wetenschappelijk instituut Statera; 2022.

57.	Giurgescu C, McFarlin BL, Lomax J, Craddock C, Albrecht A. Racial Discrimination and the Black‐White Gap in Adverse Birth Outcomes: A Review. J Midwifery Womens Health. 2011 Jul 6;56(4):362–70. doi: 10.1111/j.1542-2011.2011.00034.x.

58.	Alhusen JL, Bower KM, Epstein E, Sharps P. Racial Discrimination and Adverse Birth Outcomes: An Integrative Review. J Midwifery Womens Health. 2016 Nov 13;61(6):707–20. doi: 10.1111/jmwh.12490.

59.	Daniels KP, Valdez Z, Chae DH, Allen AM. Direct and Vicarious Racial Discrimination at Three Life Stages and Preterm Labor: Results from the African American Women’s Heart &amp; Health Study. Matern Child Health J. 2020 Nov 12;24(11):1387–95. doi: 10.1007/s10995-020-03003-4.

60.	Attanasio L, Kozhimannil KB. Patient-reported Communication Quality and Perceived Discrimination in Maternity Care. Med Care. 2015 Oct;53(10):863–71. doi: 10.1097/MLR.0000000000000411.

61.	Headen IE, Elovitz MA, Battarbee AN, Lo JO, Debbink MP. Racism and perinatal health inequities research: where we have been and where we should go. Am J Obstet Gynecol. 2022 Oct;227(4):560–70. doi: 10.1016/j.ajog.2022.05.033.

62.	Pabayo R, Ehntholt A, Davis K, Liu SY, Muennig P, Cook DM. Structural Racism and Odds for Infant Mortality Among Infants Born in the United States 2010. J Racial Ethn Heal Disparities. 2019 Dec 15;6(6):1095–106. doi: 10.1007/s40615-019-00612-w.

63.	Liu SY, Fiorentini C, Bailey Z, Huynh M, McVeigh K, Kaplan D. Structural Racism and Severe Maternal Morbidity in New York State. Clin Med Insights Women’s Heal. 2019 Jan 14;12:1179562X1985477. doi: 10.1177/1179562X19854778.

64.	Debbink MP, Bader MDM. Racial Residential Segregation and Low Birth Weight in Michigan’s Metropolitan Areas. Am J Public Health. 2011 Sep;101(9):1714–20. doi: 10.2105/AJPH.2011.300152.

65.	Salow AD, Pool LR, Grobman WA, Kershaw KN. Associations of neighborhood-level racial residential segregation with adverse pregnancy outcomes. Am J Obstet Gynecol. 2018 Mar;218(3):351.e1-351.e7. doi: 10.1016/j.ajog.2018.01.022.

66.	Willey S, Desmyth K, Truong M. Racism, healthcare access and health equity for people seeking asylum. Nurs Inq. 2022 Jan 26;29(1). doi: 10.1111/nin.12440.

67.	FitzGerald C, Hurst S. Implicit bias in healthcare professionals: a systematic review. BMC Med Ethics. 2017 Dec 1;18(1):19. doi: 10.1186/s12910-017-0179-8.

68.	Mulder L, Wouters A, Twisk JWR, Koster AS, Akwiwu EU, Ravesloot JH, et al. Selection for health professions education leads to increased inequality of opportunity and decreased student diversity in The Netherlands, but lottery is no solution: A retrospective multi-cohort study. Med Teach. 2022 Jul 3;44(7):790–9. doi: 10.1080/0142159X.2022.2041189.

69.	Clayborne EP, Martin DR, Goett RR, Chandrasekaran EB, McGreevy J. Diversity pipelines: The rationale to recruit and support minority physicians. J Am Coll Emerg Physicians Open. 2021 Feb 2;2(1). doi: 10.1002/emp2.12343.

70.	Cohen JJ, Gabriel BA, Terrell C. The Case For Diversity In The Health Care Workforce. Health Aff. 2002 Sep;21(5):90–102. doi: 10.1377/hlthaff.21.5.90.

71.	Parker RB, Stack SJ, Schneider SM, Bowman SH, Broderick KB, Brown NA, et al. Why Diversity and Inclusion Are Critical to the American College of Emergency Physicians’ Future Success. Ann Emerg Med. 2017 Jun;69(6):714–7. doi: 10.1016/j.annemergmed.2016.11.030.

**Appendix 1: National Perinatal Audit registry in the Netherlands**

*This appendix is partially based on previous descriptions of the Dutch national perinatal audit by van der Geest et al. and Rosman et al.* (26,27)*.*

During perinatal audit meetings health care providers evaluate the provided care in cases with adverse perinatal or maternal outcomes. Audit meetings take place biannually at most hospitals in the Netherlands. Each audit meeting is prepared by a local team and chaired by an independent supervisor. All perinatal health care professionals within a region are invited to audit meetings, including obstetricians, community midwives, clinical midwives, pediatricians, and obstetric nurses. Cases are discussed anonymously and involved health care providers can stay anonymous if they wish.

Which cases are discussed depends on the cases health care providers submit and whether these cases fall within one of the four audit themes. These themes change every two to three years and are determined by Perined in collaboration with experts in the field. During this study, the audit themes included late premature mortality (between 32+0 and 36+6 weeks), perinatal asphyxia (above 37+0 weeks), hyperbilirubinemia, and uterine rupture. Most cases discussed in perinatal audits fall within these themes although exceptions to this rule exist.

During an audit meeting, suboptimal factors and improvement points are constructed based on health care providers' evaluation of the cases. After reaching a consensus on the formulation of suboptimal factors and action points, these are registered in the National Perinatal audit registry. The local audit team also constructs a chronological report of each discussed case, which includes maternal characteristics, obstetric history, relevant prenatal consultations, a delivery report, and a summary of postpartum care, including maternal and neonatal follow-up. As this report is based on medical records, its completeness depends on the accuracy and thoroughness of involved health care professionals’ documentation.

Chronological reports and their corresponding suboptimal factors and action points are stored in the national Perinatal Audit registry. This registry facilitates the confidential and anonymous registration of the cases, the audit process, and its outcomes. As most local teams only discuss two cases per audit meeting, the National Perinatal Audit registry doesn’t contain all cases with adverse outcomes, but only the selection of cases discussed in local audits.

**Appendix 2: Detailed description of suboptimal factors**

Table A: Detailed description of suboptimal factors

| Suboptimal factor | Description in case report: | Specification or issues/consequences described |
| --- | --- | --- |
| Phase 1: Care seeking |  |  |
| Untimely start of antenatal care | First antenatal care appointment after 12 weeks of gestation. | Average start of antenatal care in weeks + days of pregnancy: 18+1 (range 12+4 - 33+4). |
| Missed appointments/ late arrival | One or more missed appointments with the midwife or in the hospital, or late arrival with consequences for care. |  |
| Non-compliance | Clients’ non-compliance with health care providers' advice. | Most important consequences:   - Missed or delayed diagnostics tests. - Medication not used according to prescription. - Refused induction of labor. |
| Delayed care seeking in case of alarm symptoms | More than two days delay in consulting health care provider or no consultation at all in the case of alarm symptoms or symptoms of labor. | Most common alarm symptoms with delay in care:   - Reduced fetal movements. - Stomach-aches. - Vaginal blood or fluid loss. |
| Vulnerable context | Factors in woman’s social situation that significantly impacted care-seeking behavior according to the care provider. | Most common factors:   - Teenage pregnancy. - Domestic violence. - Undocumented status. |
| Partially uncontrolled pregnancy | Interruption of antenatal care | N = 5 cases:   - Four cases of interrupted care for 10, 6, 4, and 7 weeks at the gestational age of 27, 32, 27, and 20 weeks respectively. - In one case with an unknown number of weeks of interrupted care, return to care at 36+6 weeks. |
| Lack of trust in health care providers | Cases in which patients' lack of trust was explicitly mentioned by the care provider |  |
| Phase 2: Accessibility of services |  |  |
| Language barrier | Language barrier with mention of consequences leading to suboptimal care | Consequences described:   - Inadequate/insufficient counseling for prenatal diagnostics and/or mode of birth. - Inadequate/insufficient coaching during labor. - Difficulties in interpreting patients’ symptoms. - Incomplete patient histories. - Unnecessary hospital admission. - Use of general anesthesia during an emergency cesarean. - No follow-up care postpartum provided. - Missed appointments. - Missed psychological screening. |
| Inadequate involvement of professional interpreters | At least one prenatal or post-partum consultation where:   1. Health care providers described insufficient involvement of a professional interpreter in the case report. 2. Negative consequences of a language barrier were described, but a professional interpreter was not involved. 3. No involvement of an interpreter was described in situations considered crucial for communication according to the research group, while it was described during other consultations. | Most mentioned informal interpreters, as a consequence of not working with professional interpreters:   - Clients’ partner. - Family member. - Friend. - Neighbor. - Underaged child. - An unknown man from the waiting room. |
| Transportation difficulties | Any description of problems related to the transportation of clients to care facilities. | Problems mentioned:   - No transportation available/accessible for women to reach the midwifery practice or hospital for consultations. - No transportation available/accessible during labor. - Taxi service for asylum seekers hard to reach, delayed service, or taxi divider's refusal to transport asylum seekers during labor.   Consequences of transportation difficulties:   - More home consultations. - Midwives take clients in their own cars. - Missed appointments. - Continued antenatal care with a community midwife while hospital care was indicated. - Delay in arrival at the hospital in a case with potentially life-threatening complications. |
| Transfer of care | Transfer of care between care facilities during pregnancy for reasons other than medical indication. | Reasons described:   - Relocations between asylum centers. - Women moving house. - Threatening deportation. - Living with different family members because of undocumented status. - Transfer of care on clients’ request.   Consequences of a transfer of care during pregnancy:   - Partially uncontrolled pregnancies. - Missed hospital appointments. - Missed or repeated diagnostic tests due to the incomplete or late transfer of medical records. |
| Financial barriers | Explicit mention of financial barriers to care | Consequences:   - No use of folic acid. - No uptake of postpartum care. - Not coming to appointments because of fear of costs. |
| The stress of the asylum procedure | Description of uncertainty or stress surrounding the asylum procedure | Examples:   - Asylum-seeking women with threat of deportation. - Fear of losing residence permit. |
| Phase 3: Quality of care |  |  |
| Missed or late diagnostic tests | Diagnostic tests should have been performed, however these opportunities were missed or carried out with a delay. | Most common diagnostics missed:   - Bilirubin testing after detection of neonatal jaundice. - Doppler and growth ultrasounds in case of IUGR. - Blood pressure measurements. - Fetal growth ultrasounds or CTGs in case of reduced fetal movements. - Fetal scalp blood sampling during labor. - Thompson scores and temperature management in case of perinatal asphyxia. - MRSA screening. - Diagnostics for persistent anemia. |
| Communication issues between care providers | Description or mention of communication issues between care providers or organizations involved in care for asylum seekers | Most common issues described:   - Incomplete or absent transfer of medical information between midwives and hospitals, different midwifery practices, different hospitals, midwives and general practitioners, and among staff within hospitals. - Late or absent communication from the Dutch Central Agency for the Reception of asylum seekers with regards to the deportation or relocation of asylum seekers. - Ultrasound centers or laboratories not communicating results with midwives. - Delay in consulting a university hospital in complex cases. - The lack of a case manager who keeps track of a client's care process. |
| No or late start of treatment | Treatment would have been indicated but was carried out late or not at all. | Most common examples:   - Delayed intervention during labor (most often in the form of an emergency cesarean). - Delayed phototherapy treatment of a neonate after detecting hyperbilirubinemia. - Missed opportunity for prophylactic treatment with acetylsalicylic acid, tranexamic acid, and/or calcium. - Delayed start of antibiotics in neonates with infection. |
| Other inadequate care | Inadequate care provided, not fitting into a different category | Most common issues described:   - Incorrect medication or incorrect dose of medication given. - Type of treatment obsolete/not evidence-based. - Care not according to a specific protocol for the condition. |
| Issues concerning documentation | Case report reflects issues such as incompleteness, inconsistency, or complete absence of documentation with a possible effect on care. | Most common examples:   - Inconsistent notes on case details or treatment provided. - Lack of notes on the treatment plan or care providers' considerations. - Lack of documentation regarding diagnostic examinations performed. |
| Incomplete history taking or counseling | Case report reflects incomplete history taking or counseling of the patient | Most common examples:   - No risk assessment of patient history for hyperbilirubinemia. - Incomplete medical history obtained. - No counseling concerning the mode of birth, risks of post-term birth, and induction of labor. - Insufficient counseling to ensure patients' understanding and trust. |
| Logistic or technical issues | Issues in logistics or availability of technical support | Most common issues:   - Staff short on time/high work pressure. - Shortage of staff, e.g., no gynecologist available to perform an emergency cesarean section. - No operation or labor room available. - No NICU availability. - Lab results unavailable. - Malfunctioning of CTG device. |
| Missed or late diagnosis | Medical conditions that were either missed or diagnosed late. | Missed diagnoses:   - Hyperbilirubinemia. - Uterus rupture. - IUGR. - Pre-eclampsia. - Neonatal coarctation aortae. - Anemia requiring blood transfusion. - Maternal acute fatty liver syndrome. |
| Delay in consultation or referral | The patient should have been referred or invited for an appointment after a referral | Most common issues:   - Delay between the registration of a patient at the midwifery clinic and intake consultation. - No referral for neonatal icterus on the first day after birth. - No referral to emergency care in case of alarm symptoms, such as severe vomiting in the third trimester, lack of fetal movements, and thrombocytopenia. |
| Insufficient or inadequate psychosocial care | Insufficient or inadequate care offered to clients with known psychological health problems | Most common examples include women for whom no referral to specialist care was made despite health care providers’ awareness of one of the following:   - Unsafe home situation due to domestic violence. - Mental health symptoms such as stress, depression, or anxiety. - Unwanted pregnancy. - Traumatic birth. |
| Inadequate action in case of no-show | Insufficient or inadequate action from the health care provider after multiple missed appointments without notice from the client | In all cases, this concerned missed appointments without action from health care providers, or actions were not as recommended by national guidelines on birth care for asylum seekers. |
| Insufficient monitoring during labor | Insufficient monitoring of a woman in labor or the fetal condition during labor | Most common examples:   - CTG not monitored regularly by a care provider. - Technical failure of CTG equipment. - No registration of maternal contractions. |
| Issues with postnatal maternity care | Insufficient attention of health care providers to counsel patients and/or make arrangements concerning postnatal maternity care | - Postnatal maternity care was arranged very late in pregnancy or not arranged at all. - Maternity care assistants not accepted by patients due to misunderstandings about the concept of maternity care. |
| Health care providers’ negative attitude | Negative attitudes towards clients reflected by the negative framing of patients in cases where other issues, such as communication difficulties, presumably played a role. | Examples of terms used to refer to patients by health care providers:   - ‘Uncooperative’ - 'Incapable of following instructions' - ‘Unreasonable’ - ‘Unmanageable’   In all cases, communication difficulties had been previously described, whereas no professional interpreters were involved. |
| Inadequate risk assessment | The responsible care provider should have been different based on the patient's known obstetric risk status | Most common issues:   - Care by a community midwife while hospital care was indicated. - Care in a regular hospital while care in a university hospital for complex conditions would have been indicated. |
| No placental pathology while indicated | Lack of diagnostics on placental pathology while indicated |  |

**Appendix 3: Suboptimal factors per category of adverse outcome**

Table A: Suboptimal factors per category of adverse outcome

| Suboptimal factors | Number of cases with suboptimal factors | | | | | |
| --- | --- | --- | --- | --- | --- | --- |
|  | Total | Intrauterine fetal death | Perinatal asphyxia above 37 weeks | Severe neonatal hyperbilirubinemia | Uterine Rupture | Other |
| **Total** | 53 | 14 | 15 | 12 | 7 | 7 |
| **Phase 1: Care seeking** | 43 (29+14) | 13 (7+6) | 10 (6+4) | 10 (7+3) | 6 (5+1) | 6 (6+0) |
| Untimely start of antenatal care | 22 (21+1) | 8 (7+1) | 6 (6+0) | 4 (4+0) | 4 (4+0) | 2 (2+0) |
| Missed appointments/ late arrival | 22 (19+3) | 5 (4+1) | 10 (9+1) | 5 (4+1) | 2 (2+0) | 1 (1+0) |
| Non-compliance | 20 (17+3) | 5 (4+1) | 7 (6+1) | 4 (3+1) | 2 (2+0) | 3 (3+0) |
| Misunderstanding | 10 (10+0) | 4 (0+0) | 4 (4+0) | 1 (1+0) | 2 (2+0) | 0 (0+0) |
| Patient’s choice | 2 (1+1) | 0 (0+0) | 2 (1+1) | 0 (0+0) | 0 (0+0) | 0 (0+0) |
| Un**clear** | 10 (8+2) | 2 (1+1) | 2 (2+0) | 3 (2+1) | 0 (0+0) | 3 (3+0) |
| Delayed care seeking in case of alarm symptoms | 18 (11+7) | 6 (2+4) | 4 (2+2) | 4 (4+0) | 3 (2+1) | 2 (2+0) |
| Vulnerable context | 15 (14+1) | 5 (5+0) | 3 (3+0) | 4 (3+1) | 3 (3+0) | 2 (2+0) |
| Partially uncontrolled pregnancy | 5 (5+0) | 0 (0+0) | 2 (2+0) | 1 (1+0) | 1 (1+0) | 1 (1+0) |
| Lack of trust in health care provider | 2 (1+1) | 1 (1+0) | 1 (0+1) | 0 (0+0) | 0 (0+0) | 0 (0+0) |
| **Phase 2: Accessibility of services** | 50 (42+8) | 13 (12+1) | 15 (13+2) | 10 (7+3) | 7 (4+3) | 7 (7+0) |
| Language barrier | 45 (38+7) | 9 (8+1) | 15 (13+2) | 9 (7+2) | 7 (4+3) | 7 (7+0) |
| Inadequate involvement of an official interpreter | 31 (24+7) | 7 (6+1) | 10 (8+2) | 5 (3+2) | 6 (3+3) | 5 (5+0) |
| Transportation difficulties | 12 (11+1) | 5 (5+0) | 1 (1+0) | 5 (4+1) | 0 (0+0) | 1 (1+0) |
| Transfer of care | 10 (10+0) | 2 (2+0) | 5 (5+0) | 2 (2+0) | 1 (1+0) | 1 (1+0) |
| Financial barriers | 3 (3+0) | 0 (0+0) | 0 (0+0) | 1 (1+0) | 1 (1+0) | 1 (1+0) |
| Uncertainty or stress surrounding the asylum procedure | 3 (3+0) | 2 (2+0) | 0 (0+0) | 1 (1+0) | 0 (0+0) | 0 (0+0) |
| **Phase 3: Quality of care** | 53 (24+29) | 14 (7+7) | 15 (9+6) | 12 (1+11) | 7 (2+5) | 7 (5+2) |
| Communication issues between care providers | 33 (29+4) | 8 (7+1) | 11 (11+0) | 6 (4+2) | 5 (5+0) | 4 (3+1) |
| Missed, late, or incomplete diagnostic tests | 32 (20+12) | 10 (7+3) | 7 (6+1) | 7 (0+7) | 6 (5+1) | 2 (2+0) |
| Late diagnostics after detecting neonatal jaundice | 7 (0+7) | NA | NA | 7 (0+7) | NA | NA |
| Other inadequate care | 26 (21+5) | 8 (7+1) | 4 (4+0) | 6 (3+3) | 6 (4+2) | 4 (4+0) |
| No or late start of treatment | 24 (10+14) | 6 (3+3) | 8 (3+5) | 4 (1+3) | 4 (1+3) | 3 (2+1) |
| Incomplete history taking or counseling | 24 (17+7) | 4 (4+0) | 4 (3+1) | 12 (6+6) | 4 (4+0) | 1 (1+0) |
| Issues concerning documentation | 19 (19+0) | 8 (8+0) | 5 (5+0) | 2 (2+0) | 3 (3+0) | 2 (2+0) |
| Missed or late diagnosis | 18 (5+13) | 6 (2+4) | 2 (1+1) | 6 (1+5) | 5 (2+3) | 1 (0+1) |
| Logistic or technical issues | 16 (14+2) | 2 (1+1) | 6 (5+1) | 5 (5+0) | 3 (3+0) | 1 (1+0) |
| Delay in consultation or referral | 16 (9+7) | 5 (4+1) | 2 (2+0) | 7 (2+5) | 2 (2+0) | 2 (1+1) |
| Insufficient or inadequate psychosocial care | 14 (14+0) | 5 (5+0) | 3 (3+0) | 3 (3+0) | 3 (3+0) | 1 (1+0) |
| Inadequate action in case of no-show | 8 (8+0) | 2 (2+0) | 2 (2+0) | 4 (4+0) | 0 (0+0) | 0 (0+0) |
| Health care providers’ negative attitude | 8 (8+0) | 2 (2+0) | 4 (4+0) | 0 (0+0) | 3 (3+0) | 0 (0+0) |
| Insufficient monitoring during labor | 7 (5+2) | 1 (1+0) | 5 (3+2) | 0 (0+0) | 2 (2+0) | 1 (1+0) |
| Issues with postnatal maternity care | 6 (4+2) | 0 (0+0) | 1 (1+0) | 5 (3+2) | 0 (0+0) | 0 (0+0) |
| Inadequate risk assessment | 4 (3+1) | 2 (1+1) | 1 (1+0) | 0 (0+0) | 0 (0+0) | 1 (1+0) |
| No placental pathology while indicated | 4 (4+0) | 0 (0+0) | 2 (2+0) | 0 (0+0) | 1 (1+0) | 1 (1+0) |

Numbers are presented as: Number of cases (minor+major).

1. When we use the term ‘refugee’ without further specification we refer to both refugees with a residence permit, asylum seekers whose claim for asylum is still pending, and undocumented migrants.
2. In this study, the term ‘woman’, also refers to individuals with a uterus who are not woman identified, including trans and non-binary individuals.
